# Body dysmorphic disorder and self-esteem: a meta-analysis

**DOI:** 10.1186/s12888-021-03185-3

**Published:** 2021-06-15

**Authors:** Nora Kuck, Lara Cafitz, Paul-Christian Bürkner, Laura Hoppen, Sabine Wilhelm, Ulrike Buhlmann

**Affiliations:** 1 Department of Psychology and Sport Science, University of Münster, Münster, North Rhine-Westphalia Germany; 2 Cluster of Excellence SimTech, University of Stuttgart, Stuttgart, Baden-Württemberg Germany; 3 Department of Psychiatry, Massachusetts General Hospital / Harvard Medical School, Boston, MA USA

**Keywords:** Body dysmorphic disorder, Body image, Self-esteem, Appearance concerns, Meta-analysis

## Abstract

**Objective:**

Body dysmorphic disorder (BDD) is associated with low self-esteem. The aim of this meta-analysis was to examine the strength of the cross-sectional relationship between BDD symptom severity and global self-esteem in individuals with BDD, mentally healthy controls, community or student samples, and cosmetic surgery patients. Moreover, the role of depressive symptom severity in this relationship and other moderating factors were investigated.

**Methods:**

A keyword-based literature search was performed to identify studies in which BDD symptoms and global self-esteem were assessed. Random effects meta-analysis of Fisher’s z-transformed correlations and partial correlations controlling for the influence of depressive symptom severity was conducted. In addition to meta-analysis of the observed effects, we corrected the individual correlations for variance restrictions to address varying ranges of BDD symptom severity across samples.

**Results:**

Twenty-five studies with a total of 6278 participants were included. A moderately negative relationship between BDD symptom severity and global self-esteem was found (*r* = −.42, *CI* = [−.48, −.35] for uncorrected correlations, *r* = −.45, *CI* = [−.51, −.39] for artifact-corrected correlations). A meta-analysis of partial correlations revealed that depressive symptom severity could partly account for the aforementioned relationship (*pr* = −.20, *CI* = [−.25, −.15] for uncorrected partial correlations, *pr* = −.23, *CI* = [−.28, −.17] for artifact-corrected partial correlations). The sample type (e.g., individuals with BDD, mentally healthy controls, or community samples) and diagnosis of BDD appeared to moderate the relationship only before artifact correction of effect sizes, whereas all moderators were non-significant in the meta-analysis of artifact-corrected correlations.

**Conclusions:**

The findings demonstrate that low self-esteem is an important hallmark of BDD beyond the influence of depressive symptoms. It appears that negative evaluation in BDD is not limited to appearance but also extends to other domains of the self. Altogether, our findings emphasize the importance of addressing self-esteem and corresponding core beliefs in prevention and treatment of BDD.

**Supplementary Information:**

The online version contains supplementary material available at 10.1186/s12888-021-03185-3.

## Introduction

Body dysmorphic disorder (BDD) is characterized by a preoccupation with perceived appearance defects and repetitive behaviors intended to hide, fix or check them. The perceived flaws are not observable or only appear minimal to others. Affected individuals may excessively check their body areas of concern, seek reassurance, camouflage or groom, compare their own physical appearance to that of others, exercise to the point of injury, or even seek cosmetic surgery [1]. The symptoms frequently lead to marked impairment in social functioning and reduced quality of life [2].

In general, BDD is associated with low self-esteem [3]. Rosenberg defined self-esteem as one’s positive or negative attitudes towards the self. Accordingly, persons may have favourable or unfavourable opinions about themselves and self-esteem is an overall evaluation of one’s value [4]. Thus, the question arises how strongly the negative evaluation in the domain of physical appearance in BDD is accompanied by general feelings of unworthiness and a low self-esteem. Several studies have investigated self-esteem in BDD (e.g., [3, 5, 6]). The samples comprised clinical samples (e.g., [7]), combined samples of patients and healthy control participants (e.g., [8]), non-clinical community (e.g., [9, 10]) or student samples (e.g., [11–13]). Moreover, data on self-esteem and BDD symptoms in cosmetic surgery settings have been collected (e.g., [14–16]). Altogether, more pronounced BDD symptoms were related to lower self-esteem in these studies. However, the reported effect sizes varied from *r* = − .04 to *r* = − .52, or *d* = 0.66 to *d* = 2.26. In addition, various authors assessed BDD symptoms and self-esteem but did not report effect sizes, and so far, no meta-analysis or review has systematically analyzed and integrated these studies.

A frequent comorbid disorder in BDD is major depression [17]. Summers et al. demonstrated the interconnectedness of BDD symptoms and depressive symptoms in a network analysis of BDD and major depressive disorder [18]. Elevated levels of depressive symptoms were found in adolescents with high appearance anxiety [19]. This shows that, regardless of the diagnostic categories, BDD and depressive symptoms tend to co-occur. Moreover, depression is linked to low self-esteem [20]. Feelings of worthlessness are among the diagnostic criteria for major depression [1]. According to a meta-analysis by Sowislo and Orth, low self-esteem represents a risk factor for depressive symptoms rather than a consequence [21]. Still, low self-esteem and depressive symptoms might reciprocally affect each other [20]. The connection of depressive symptoms to self-esteem and BDD may have consequences for the relationship between BDD symptoms and self-esteem. More precisely, the co-occurrence of BDD symptoms and low self-esteem may either be specific to BDD or may be caused by high levels of comorbid depressive symptoms. In this regard, Cerea et al. already pointed to the relevance of clarifying the relationship between BDD and self-esteem [9]. So far, only two studies reported partial correlations and suggested that depressive symptoms might contribute to the relationship between BDD symptoms and self-esteem. A study by K. A. Phillips et al. revealed a zero-order correlation of *r* = − .38 and a partial correlation of *pr* = −.16 [3]. Bartsch et al. found an uncontrolled correlation of *r* = − .48 and a partial correlation of *pr* = −.32 [22]. Besides, several studies measured depressive symptoms alongside with BDD symptoms and self-esteem but did not provide a partial correlation. Analyzing these studies with meta-analytic techniques and gathering corresponding effect sizes can shed light on the role of depressive symptoms.

Another relevant question is whether the strength of the relationship between BDD symptoms and self-esteem varies systematically between different subgroups. On the one hand, low self-esteem might particularly act as a risk factor for BDD in certain groups such as adolescence. Adolescence is a developmental phase in which body image concerns are common [23]. BDD most frequently begins in this period [24]. Also, adolescence is characterized by declining self-esteem [25–27]. Furthermore, decreased self-esteem appears to be strongly related to dysmorphic concern in adolescents [28]. Thus, if low self-esteem represented a risk factor for BDD, it could have a more severe impact in a vulnerable period such as adolescence. On the other hand, BDD symptoms might result in lower self-esteem in adolescence and young adulthood than in middle and old age. The concept of contingent self-esteem refers to the degree to which self-esteem depends on achievements and feedback in different domains such as appearance, academic success, relationships, or virtue [29]. A study by Meier et al. suggested that self-esteem might become less contingent on interpersonal conflicts across the life course [25]. If contingent self-esteem also decreased in other domains, a preoccupation with perceived defects in appearance might have a larger effect on self-esteem in adolescence and young adulthood compared to middle and old age. Further, some studies found that women tend to have more contingent self-esteem than men, particularly in the domain of appearance [25, 30]. Hence, BDD symptoms might possibly affect self-esteem more strongly in women than in men. Alternatively, it is possible that the effects of appearance concerns on self-esteem are stronger in individuals with (vs. without) a clinical diagnosis of BDD given that - according to our clinical observation - individuals with clinical BDD build their self-esteem predominantly on how they look. So far, there has been a lack of longitudinal studies on BDD symptoms and self-esteem, and therefore we do not know whether low self-esteem could cause BDD. Also, the current studies did not investigate moderators of the cross-sectional relationship between BDD symptoms and self-esteem. However, meta-analytic studies allow for a closer investigation of systematic variation in effect sizes. Thus, insights on the influence of age, gender, or sample type on the relationship between BDD symptoms and self-esteem can be gained.

In summary, the aims of the current meta-analysis were as follows:

1. Examine the strength of the cross-sectional relationship between BDD symptom severity and global self-esteem in BDD patients, healthy controls, community or student samples, and cosmetic surgery patients.
2. Investigate whether the aforementioned relationship between BDD symptom severity and self-esteem persists beyond the influence of depressive symptoms.
3. Explore potential systematic differences in the magnitude of the correlations regarding participants’ mean age, percentage of females, the sample type (e.g., student sample or BDD patients), the diagnostic method (self-report versus clinician-administered measures of BDD symptoms), and BDD diagnosis (whether BDD was diagnosed by a clinician prior to or during study participation).

Altogether, the three research questions could further our understanding of associated features in BDD and offer valuable insights for the prevention and treatment of BDD.

## Methods

A preprint of the manuscript was uploaded to psyarxiv (https://psyarxiv.com/). The extracted data used for the meta-analysis are available at our Open Science Framework (OSF) data repository (https://osf.io/z52fc/). A PRISMA checklist concerning the documentation of the meta-analysis can be retrieved in the Appendix (Additional file 1) [31]. The meta-analysis was not pre-registered.

### Study selection

Studies were selected if they fulfilled the following eligibility criteria. BDD symptom severity had to be measured with a questionnaire or interview that captures symptoms as described in the fifth or fourth edition of the *Diagnostic and Statistical Manual of Mental Disorders*, DSM-5 or DSM-IV [1, 32]. This comprised detailed measures of BDD symptom severity as well as shorter screening measures for BDD symptoms. Alternatively, categorial diagnostic measures of BDD based on DSM-IV or DSM-5 were also considered. Hence, the Yale-Brown Obsessive Compulsive Scale for Body Dysmorphic Disorder (BDD-YBOCS) [33], the self-report and clinician-administered versions of the Body Dysmorphic Disorder Examination (BDDE) [34], the Body Dysmorphic Symptoms Inventory (Fragebogen körperdysmorpher Symptome; FKS) [35], the Questionario sul Dismorfismo Corporeo (QDC) [36], the Dysmorphic Concern Questionnaire (DCQ) [37], the Body Dysmorphic Disorder Questionnaire (BDDQ) [38], and the Body Dysmorphic Disorder Diagnostic Module (BDD-DM) [39] were included in this meta-analysis. Measures of body image or body dissatisfaction were excluded. Also, measures which specifically address muscle dysmorphia were not included, as we intended to investigate BDD symptoms in general and because of the overlap between muscle dysmorphia and eating disorders. This meta-analysis relied on the definition and operationalization of self-esteem by Rosenberg [4]. Thus, self-esteem needed to be assessed via the Rosenberg Self-Esteem Scale (RSES), the most widely used self-report measure for global self-esteem [4]. For inclusion in the meta-analysis of partial correlations, studies were required to use a questionnaire or interview for the assessment of depressive symptom severity. The Beck Depression Inventory (BDI) [40–42], the Hamilton Depression Rating Scale (HAMD) [43], the depression subscale of the Depression Anxiety Stress Scales (DASS) [44], the depression subscale of the Hospital Anxiety and Depression Scale (HADS) [45], the depression subscale of the Symptom Checklist-90 (SCL-90) [46], and the Patient Health Questionnaire-9 Depression module (PHQ-9) [47] were used in the studies.

Clinical, subclinical, and non-clinical samples were examined. Studies could target BDD patients, mentally healthy control participants, students, community persons, and cosmetic surgery patients. Participants were allowed to have secondary comorbid mental disorders. However, samples with another primary mental disorder (e.g., eating disorders, social anxiety disorder) were excluded. Studies that were recruited according to the presence or absence of a physical condition (e.g., rheumatic arthritis, obesity) were not included in this analysis. Also, samples that were selected according to related factors (e.g., body dissatisfaction) were not considered. No restrictions concerning age or gender of the sample were applied. Studies could be designed as correlational surveys or intervention studies. Since we investigated the cross-sectional relationship, data on all our variables of interest had to be collected at a single measurement point. In the case of more than one measurement point, baseline measures were analyzed. Case studies were omitted. For inclusion, manuscripts were required to be written in English or German.

Several sources were used to identify relevant studies. The databases *PubMed*, *PsycInfo*, *PsycArticles*, *Medline*, *Web of Science*, *Psyndex*, and *Dissertation Abstracts International* were searched for eligible studies. Furthermore, ongoing trials were found in the http://ClinicalTrials.gov registry, the *Cochrane Central Register of Controlled Trials* (*CENTRAL*), the *WHO International Clinical Trials Registry Platform* (*ICTRP*), and the *ISRCTN* registry. We also tried to obtain unpublished data by searching *OpenGrey* (http://www.opengrey.eu). The keyword-based literature search was carried out by the second author in April 2017. Subsequently published or registered studies were identified in January 2019, August 2019, and in May 2020. The following search term was applied: (body dysmorphic AND self-esteem) or (dysmorphophobia AND self-esteem) or (dysmorphophobic AND self-esteem) or (body dysmorphic AND self-worth) or (dysmorphophobia AND self-worth) or (dysmorphophobic AND self-worth). The corresponding German search terms were: (körperdysmorphe AND Selbstwert) or (Dysmorphophobie AND Selbstwert) or (dysmorphophobe AND Selbstwert). Additionally, 24 well-known researchers in the field of BDD were contacted for unpublished studies in September 2019.

In a first step, the abstracts of identified studies were screened. The abstract screening of studies which were published after April 2017 was performed by two research assistants. The abstracts were required to suggest that BDD symptoms and self-esteem were captured in the study. Subsequently, a full text assessment was conducted by the second author (or a research assistant for studies with dates of publication after April 2017) according to the eligibility criteria described above.

### Data collection

A coding scheme for extraction of relevant data was developed. The coding scheme contained the following information: First, the sample was described with regard to the number of participants (in total and in the subgroups), clinical status, age, sex, education, ethnicity, sample type (e.g., students, cosmetic surgery patients), comorbidities, and other study-specific inclusion criteria (e.g., a certain cut-off on a BDD questionnaire). Second, the assessment of BDD symptom severity was specified. The interview or questionnaire used to examine BDD symptoms, diagnostic criteria, the diagnostic method (self-report vs. clinician-administered), as well as means and standard deviations of the diagnostic measure in the sample were coded. Additionally, the range of BDD symptom severity (e.g., only clinical participants) and whether the study compared two extreme groups (e.g., BDD patients versus healthy controls) were rated. Third, mean and standard deviation of the RSES in the total sample were gathered. Fourth, information on the assessment of depressive symptoms was collected. This included the measure for depressive symptom severity, the applied diagnostic criteria, the diagnostic method, as well as mean and standard deviation of the measure for depressive symptoms. Fifth, the reported effect size data were compiled. Preferably, the correlations between BDD symptom severity and self-esteem, between BDD symptom severity and depressive symptom severity, and between self-esteem and depressive symptom severity were gathered. Additionally, we coded whether the correlation was reported in the study or obtained by the authors afterwards. The type of correlation and the number of participants, for whom the correlation was calculated, were also coded. Alternatively, Cohen’s d for the difference in self-esteem and depressive symptoms of participants with BDD compared to participants without BDD were entered. If Cohen’s d was not reported, the mean and standard deviation of self-esteem and depressive symptom severity, and the number of participants in each comparison group were collected.

Data were coded independently by the first and second author. Interrater agreement was 97% and consensus was achieved after discussion of divergent coding. If studies did not report all data that were needed for the meta-analysis, authors were asked for the missing information. Altogether, 30 authors were contacted (concerning 35 studies) and 17 authors provided the required information (for 20 studies).

The effect sizes in the individual studies might have been subject to bias. We considered the selection of the sample (e.g., clinical BDD patients versus non-clinical students) and the diagnostic method for assessing BDD symptoms (self-report versus clinician-administered) as possible sources of bias. Consequently, these aspects were included in our coding scheme and controlled for in moderator analysis. Furthermore, we dealt with potential selective reporting by contacting all authors of studies which assessed our variables of interest without reporting an effect size for the relationship between BDD symptoms and self-esteem.

### Data analysis

Effect sizes for the relationship between BDD symptom severity and self-esteem were calculated in three ways depending on the level of measurement of BDD symptom severity. For the majority of studies (*k* = 21), Fisher’s z transformed Pearson correlations between BDD symptom severity and self-esteem were analyzed. If effect sizes could not be based on a continuous measure of BDD symptom severity, we either used the pointbiseral correlation (*k* = 1) between BDD (coded 1 for BDD and 0 for healthy controls) and self-esteem or Cohen’s d (*k* = 1) which was transformed to Fisher’s z [48, 49]. In this case Cohen’s d described the difference in mean self-esteem between participants with BDD compared to participants without BDD. This categorial effect size is not based on the individual values of participants but rather on the group means. Thus, it mirrors the relationship between BDD symptom severity and self-esteem on a less precise group level. Nevertheless, we preferred to integrate these categorial effect sizes in the meta-analysis to achieve an extensive overview of the field and to avoid complete loss of the information. Two studies [12, 50] followed an ordinal approach and reported correlations between the number of items endorsed on the BDDQ and self-esteem. As this represents a gain in information compared to mere nominal data, this procedure was applied for studies which used the BDDQ.

If possible, an effect size for the total sample (instead of separate effect sizes for the subgroups) was gathered. Still, samples with varying ranges of BDD symptom severity were examined. In some cases, this may have caused underestimation of the true effect, whereas in others the magnitude of the relationship might have been overestimated [51]. Restriction of range in samples with reduced variance of BDD symptom severity (e.g., only clinical BDD participants) may have led to underestimation of the true effect. Enhancement of range and corresponding overestimation of effect sizes may have been produced by comparison of extreme groups (BDD patients versus healthy controls). A meta-analysis without artifact correction was conducted to describe the actual observed effects. Additionally, we attempted to correct for the artifacts. Thereby, we intended to achieve an estimate of the effect scaled on the general population without variance restrictions. For this purpose, studies with potentially restricted or enhanced range of BDD symptom severity were identified on the basis of theoretical assumptions concerning the sample. The individual correlations of these studies were adjusted before conducting a meta-analysis using standard corrections for variance restrictions [52]. For the adjustment, an estimate of the standard deviation of the BDD symptom severity measure in the general population was used and applied to all studies included. If possible, this was drawn from studies with large community samples.

For the calculation of partial correlations between BDD symptom severity and self-esteem controlling for depressive symptom severity, Pearson correlations between BDD symptom severity and depressive symptom severity, as well as between self-esteem and depressive symptom severity were conducted and preprocessed in the same manner as described above. The partial correlations controlling for depressive symptom severity were also Fisher’s z transformed for a subsequent meta-analysis. A meta-analysis of (z-transformed) partial correlations was also conducted with and without artifact correction.

A random effects meta-analysis was chosen to account for heterogeneity in effect sizes across studies. The computation was performed in *R* [53] using the *metafor* package [54]*.* For the assessment of effect size variability I^2^ and τ were used. A moderator analysis was conducted to examine the influence of participants’ mean age, percentage of females, sample type, diagnostic method, and BDD diagnosis on effect sizes. An alpha level of α = .05 was applied. To visualize a potential publication bias, we created funnel plots.

## Results

### Study characteristics

**Fig. 1:**
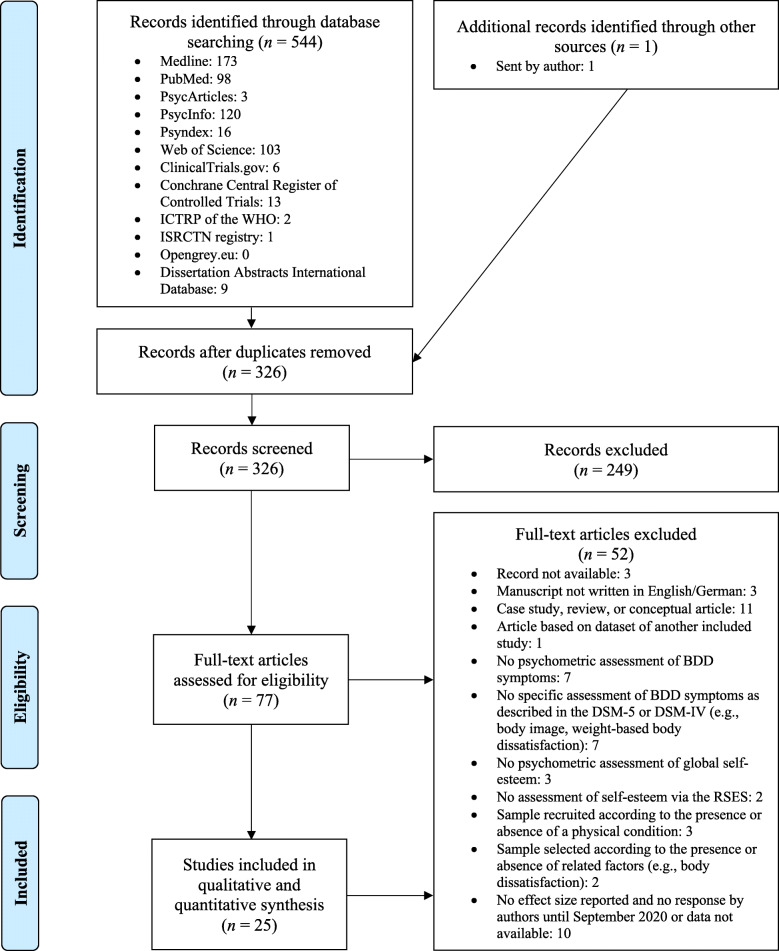
PRISMA flow diagram illustrating the process of study selection

The process of study selection with the number of records screened and excluded at each stage is presented in the PRISMA flow diagram in Fig. 1 [31]. Altogether, 25 studies (and 27 effect sizes) with a total number of 6278 participants were included in the meta-analysis. The mean age was 26.35 with a mean percentage of females of 69.62%. Regarding the sample type, four samples were drawn from individuals with clinical BDD (*n* = 239), three from mentally healthy control participants and individuals with clinical BDD (*n* = 128), and five from cosmetic surgery settings (*n* = 614). Further, nine student samples (*n* = 3463), two community samples (*n* = 423), and three community samples with large proportions of students (*n* = 1310) were analyzed. For nine studies BDD was diagnosed by a clinician either prior to or during study participation. Twelve effect sizes were based on clinician-rated measures of BDD symptoms whereas 14 relied on self-report measures (for one study no precise information was available whether the BDD-YBOCS was administered by a clinician or applied as a self-report questionnaire). Seventeen studies assessed depressive symptoms and could be included in the meta-analysis of partial correlations. Table 1 provides an overview of the study characteristics and effect sizes which were extracted from the studies.

**Table 1:** Study characteristics and effect sizes

Authors, Year	Sample type	N	Age M (SD)	Females (%)	BDD measure	BDD M (SD)	BDD diagnosis	Measure of depressive symptoms	r	pr
Ahmadpanah et al., 2019 [13]	Student	350	24.19 (3.71)	76.90	BDD-YBOCS	12.34 (5.81)	no		−.04	
Bartsch, 2007 [22]	Student	619	26.14	72.70	DCQ	13.24	no	DASS	−.48	−.29
Baykal et al., 2015 [16]	Cosmetic surgery	56	27.88 (8.62)	55.36	BDDE-SR	46.57 (36.67)	no		−.52	
Bohne, Keuthen, et al., 2002 [50]	Student	91	21.00 (2.40)	82.20	BDDQ	1.29 (1.22)	no	BDI-I	−.31	−.14
Bohne, Wilhelm, et al., 2002 [12]	Student	133	22.00 (3.50)	73.70	BDDQ	0.78 (1.21)	no	BDI-I	−.26	−.10
Boroughs et al., 2010 [55]	Student	1041	20.95 (4.24)	66.95	BDDE-SR	45.58 (28.26)	no		−.32	
Buhlmann et al., 2008 [6]	BDD / HC	55	23.98 (7.57)	81.82	FKS	34.41 (11.19)	yes	BDI-I	−.69	−.29
Buhlmann et al., 2009 [5]	BDD	42	28.21 (9.06)	90.48	BDD-YBOCS	15.14 (6.52)	yes	BDI-I	−.35	−.18
Cerea et al., 2018 [9]	Community / student	615	30.51 (13.26)	69.40	QDC	95.86 (38.46)	no	DASS-21	−.52	−.36
Cerea et al., 2021 [56]	Student	20	22.00 (1.52)	100.00	QDC	144.65 (12.47)	no	DASS-21	-.06	.02
Dietel et al., 2018 [11]	Student	112	22.45 (3.10)	73.21	FKS	9.72 (6.45)	no	BDI-II	−.27	−.10
Dogan & Yassa, 2019 [57]	Cosmetic surgery	71	32.00 (9.01)	100.00	BDD-YBOCS	22.99 (8.06)	yes		−.35	
Dowling et al., 2010 [14]	Cosmetic surgery	333	36.45 (11.52)	89.79	DCQ	8.20 (4.28)	no	HADS	−.36	−.18
Grocholewski et al., 2013 [7]	BDD	23	30.96 (11.42)	65.22	BDD-YBOCS	29.00 (5.89)	yes	BDI-I	−.54	−.22
Hartmann et al., 2014 [8]	BDD / HC	45	29.40 (12.13)	71.11	BDD-YBOCS	15.31 (14.49)	yes	BDI-II	−.70	−.43
Jorge et al., 2008 [58]	Cosmetic surgery	33	38.10	100.00	BDDE	80.80	no		−.23	
Labuschagne et al., 2010 [48]	BDD / HC	28	33.00 (13.17)	64.29	BDD-DM		yes	BDI-II	-.64^d^	−.15
Mulkens et al., 2012 [15]	Cosmetic surgery	121	45.40 (11.80)	100.00	BDDE-SR	26.80 (20.90)	no	SCL-90	−.39	−.16
B. Phillips et al., 2011 [10]	Community / student	194	24.70 (9.34)	76.29	BDD-YBOCS self-report^a^	11.03 (7.38)	no	DASS-21	−.43	−.12
K. A. Phillips et al., 2004 [3]	BDD	92	32.10 (10.50)	71.00	BDD-YBOCS	31.10 (5.80)	yes	HAMD	−.38	−.14
Rosen & Ramirez, 1998 [49]	^b^	101	34.09	60.40	BDDE	72.23	yes		-.69^c^	
Rosen & Reiter, 1996 [34]	BDD	82	34.67	71.95	BDDE	94.40	yes		−.33	
Rosen & Reiter, 1996 [34]	Student	295	18.72	55.60	BDDE	34.25	no		−.50	
Rosen & Reiter, 1996 [34]	Community	140	40.55	55.00	BDDE	22.27	no		−.49	
Sadighpour et al., 2019 [59]	Student	802	20.79 (2.10)	62.30	BDD-YBOCS^a,e^	10.80 (5.70)	no		−.38	
Schmidt & Martin, 2019 [60]	Community / student	501	31.20 (11.70)	81.24	DCQ	7.76 (4.70)	no	PHQ-9	−.43	−.13
Wang et al., 2014 [61]	Community	283	23.66	24.38	BDDE-SR	32.62 (17.40)	no	BDI-I	−.35	−.19

Student, student sample; Cosmetic surgery, cosmetic surgery sample; Community, community sample; BDD, clinical BDD sample; BDD / HC, sample of individuals with BDD and healthy controls; Community / student, community sample with large proportion of students; BDD-YBOCS, Yale-Brown Obsessive-Compulsive Scale Modified for Body Dysmorphic Disorder; DCQ, Dysmorphic Concern Questionnaire; BDDE-SR, Body Dysmorphic Disorder Examination - Self Report; BDDE, Body Dysmorphic Disorder Examination; BDDQ, Body Dysmorphic Disorder Questionnaire; FKS, Fragebogen körperdysmorpher Symptome; QDC, Questionario sul Dismorfismo Corporeo; BDD-DM, Body Dysmorphic Disorder Diagnostic Module; DASS, depression subscale of the Depression Anxiety Stress Scales; DASS-21, depression subscale of the modified, shorter 21-item version of the Depression Anxiety Stress Scales; BDI-I, Beck Depression Inventory-I; BDI-II, Beck Depression Inventory-II; HADS, depression subscale of the Hospital Anxiety and Depression Scale; SCL-90, depression subscale of the Symptom Checklist-90; HAMD, Hamilton Depression Rating Scale; PHQ-9, depression module of the Patient Health Questionnaire; r, uncorrected correlation between BDD symptom severity and global self-esteem; pr, uncorrected partial correlation between BDD symptom severity and global self-esteem controlling for depressive symptom severity

^a^ Ten out of 12 items were used in these studies

^b^ This study was not included in the moderator analysis for the sample type as it compared a clinical BDD sample to a non-clinical sample in which the absence or presence of mental disorders were not verified

^c^ Transformed from d to r

^d^ Pointbiseral correlation between BDD (coded 1 for BDD and 0 for healthy controls) and self-esteem

^e^ This study was not included in the moderator analysis for the diagnostic method as it was not clearly specified if the self-report or clinician-administered version of the BDD-YBOCS were used

### Meta-analysis of zero-order correlations

**Fig. 2:**
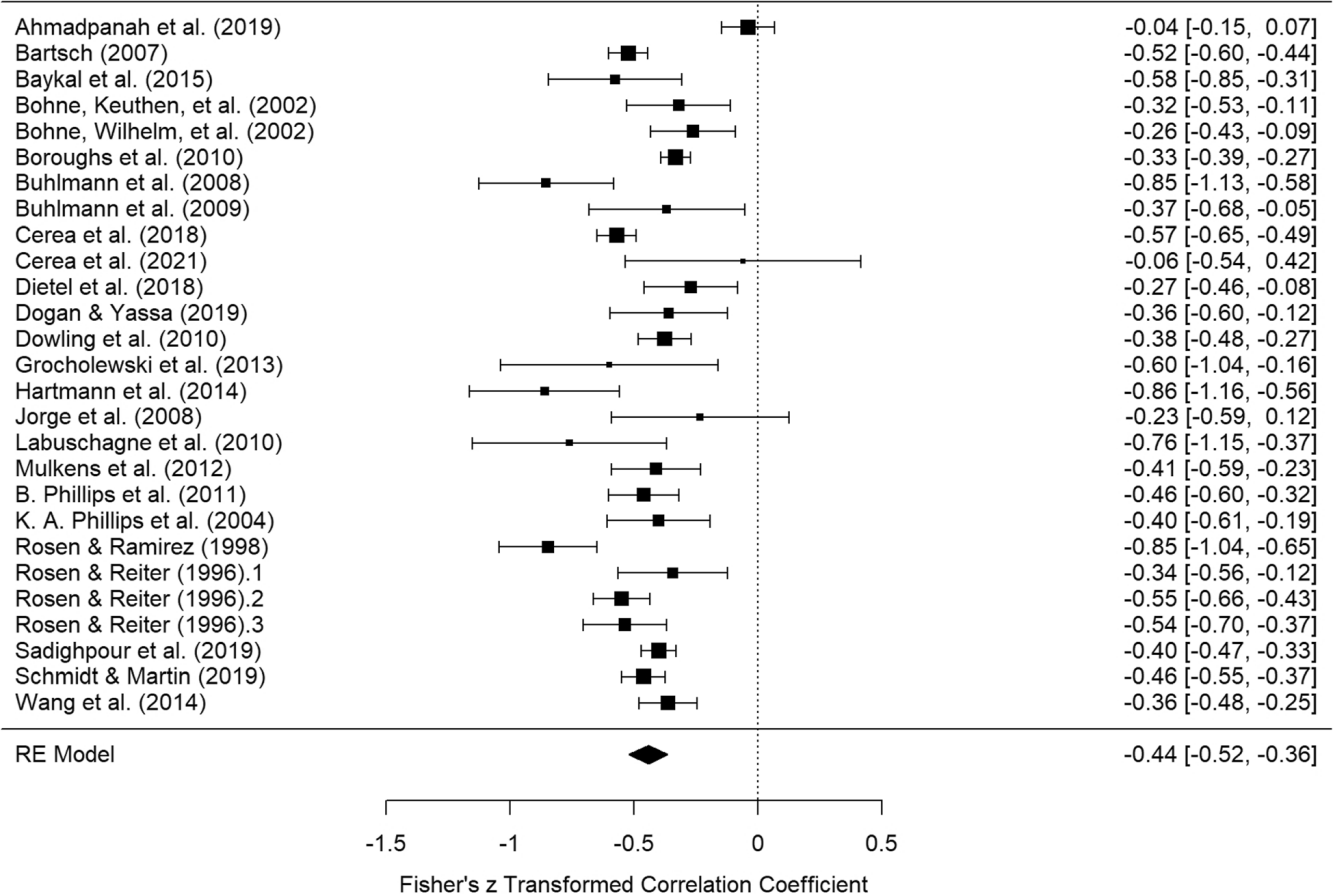
Forest plot of Fisher’s z-transformed correlations between BDD symptom severity and self-esteem

The meta-analysis of uncorrected zero-order correlations between BDD symptom severity and self-esteem yielded an overall effect size of *r* = −.42, *CI* = [−.48, −.35]. The Fisher’s z-transformed effect estimates and confidence intervals for the individual studies as well as the Fisher’s z-transformed overall effect size are illustrated in Fig. 2. With regard to heterogeneity, *I*^*2*^ amounted to 85.87% and τ was .17, indicating substantial variability of effect sizes.

**Fig. 3:**
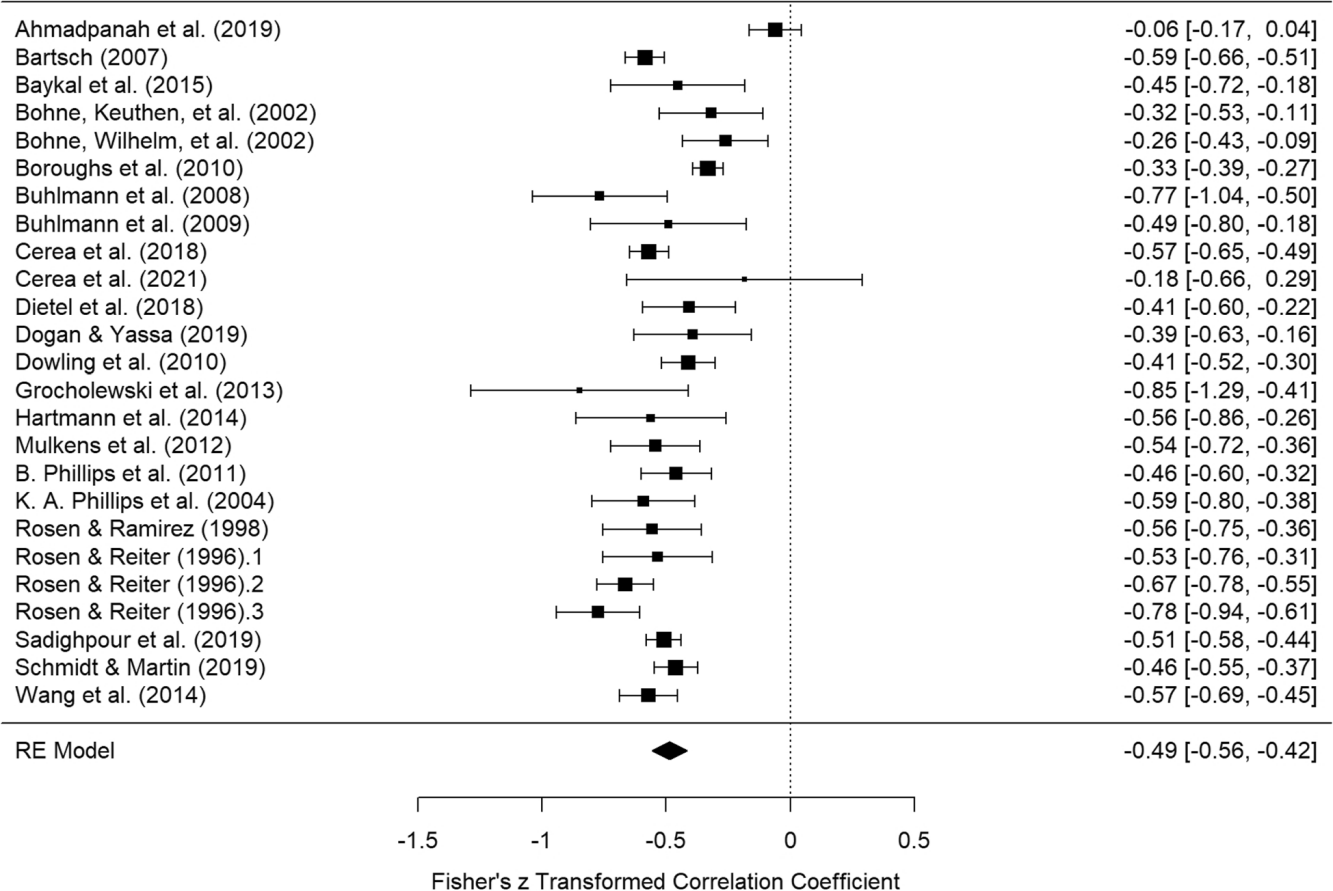
Forest plot of Fisher’s z-transformed correlations between BDD symptom severity and self-esteem (corrected for variance restriction and enhancement of BDD symptom severity)

When correcting for variance restriction and enhancement of BDD symptom severity, a mean weighted correlation of *r* = −.45, *CI* = [−.51, −.39] was observed. The artifact-corrected Fisher’s z-transformed zero-order correlations and the corresponding overall effect size estimate are visualized in Fig. 3. The *I*^*2*^ of 82.38% and τ = .14 implied considerable heterogeneity. The standard deviation estimates for the BDD symptom severity measures which were used for artifact correction can be found in the appendix (Additional file 2).

### Meta-analysis of partial correlations

**Fig. 4:**
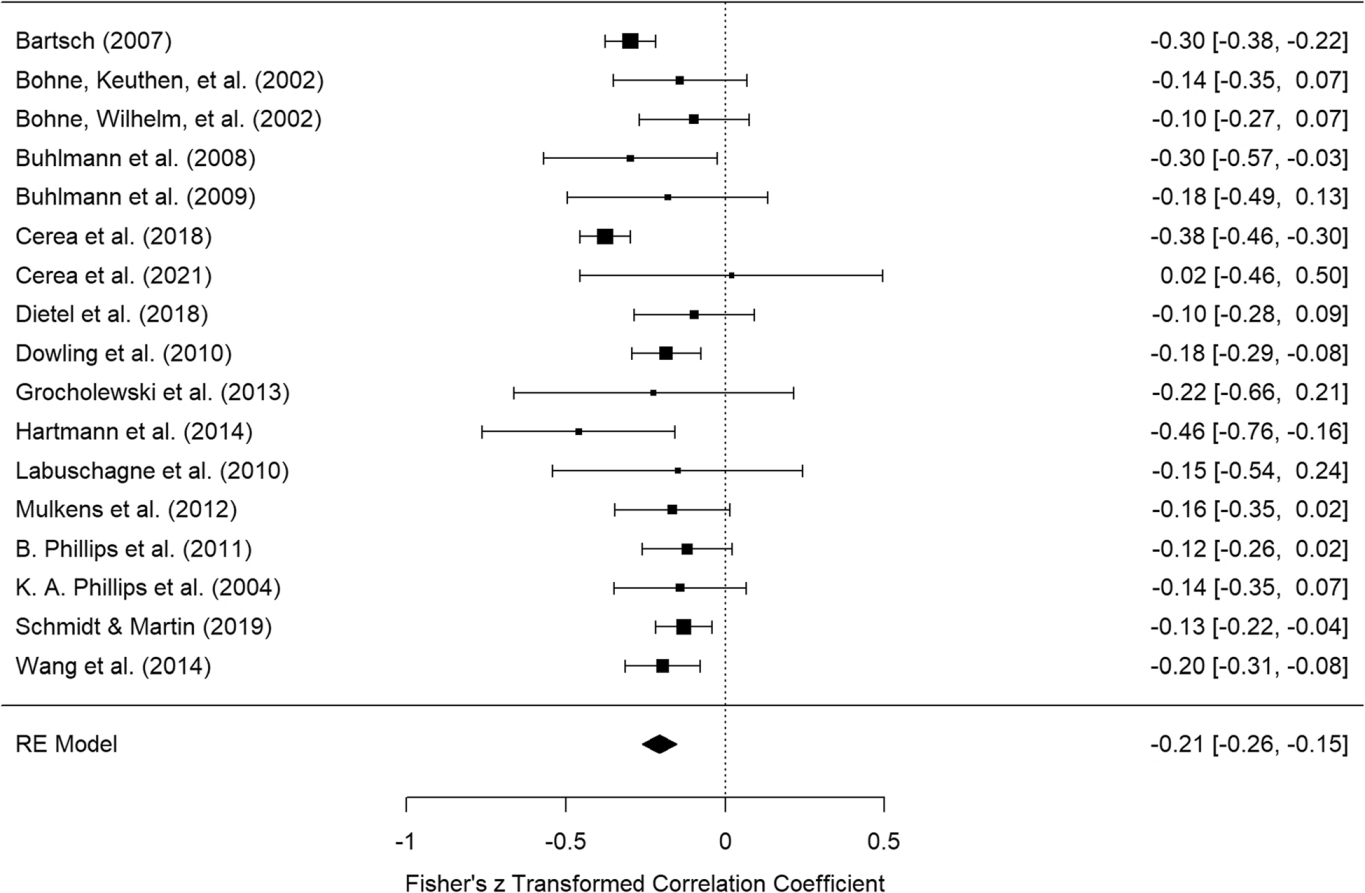
Forest plot of Fisher’s z-transformed partial correlations between BDD symptom severity and self-esteem controlling for depressive symptom severity

In the meta-analysis of uncorrected partial correlations between BDD symptom severity and self-esteem controlling for depressive symptom severity a mean weighted effect size of *pr* = −.20, *CI* = [−.25, −.15] was achieved. The forest plot of Fisher’s z-transformed partial correlations and confidence intervals for the individual studies and the total estimate are displayed in Fig. 4. Investigation of heterogeneity resulted in *I*^*2*^ = 37.28% and τ = .06.

**Fig. 5:**
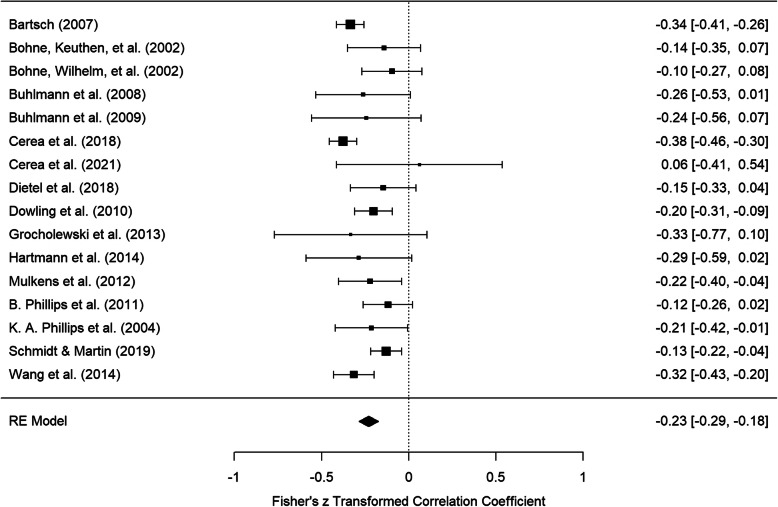
Forest plot of Fisher’s z-transformed partial correlations between BDD symptom severity and self-esteem controlling for depressive symptom severity (corrected for variance restriction and enhancement of BDD symptom severity)

Basing the meta-analysis on the artifact-corrected partial correlations revealed a mean weighted effect size of *pr* = −.23, *CI* = [−.28, −.17]. Fisher’s z transformed coefficients and confidence intervals are presented in Fig. 5. This analysis produced an *I*^*2*^ of 40.33% and τ = .06.

### Moderator analysis

The results of the moderator analysis for the meta-analysis of uncorrected zero-order correlations are presented in Table 2. The mean age of the sample, the percentage of females, and the diagnostic method did not show a significant influence on the magnitude of effect sizes in any of the analyses. The sample type turned out to be a significant moderator in the meta-analysis of uncorrected zero-order correlations (*F* (3, 22) = 4.83, *p* < .01). The weighted effect size estimates were *z* = −.40, *CI* = [−.58, −.22] for clinical BDD samples, *z* = −.83, *CI* = [− 1.06, −.60] for combined samples of mentally healthy control participants and individuals with clinical BDD, *z* = −.39, *CI* = [−.46, −.32] for student and community samples (which were analyzed as one category in the moderator analysis), and *z* = −.40, *CI* = [−.54, −.25] for the cosmetic surgery samples. The effect sizes for combined samples of clinical BDD and mentally healthy control participants differed significantly from the clinical BDD samples when contrasted in a dummy-coded moderator analysis (cf., Table 2). However, the moderation effect of the sample type was no longer significant for the artifact-corrected zero-order correlations. The weighted effect size estimates for the artifact-corrected zero-order correlations amounted to *z* = −.59, *CI* = [−.80, −.38] for clinical BDD samples, *z* = −.67, *CI* = [−.97, −.37] for combined samples of mentally healthy control participants and individuals with clinical BDD, *z* = −.46, *CI* = [−.55, −.37] for student and community samples, and *z* = −.45, *CI* = [−.63, −.27] for the cosmetic surgery samples. Regarding the partial correlations, the moderation effect of the sample type was no longer significant. Even more so, effect sizes for the different sample types were very much aligned after artifact correction (*z* = −.24, *CI* = [−.45, −.04] for clinical BDD samples, *z* = −.27,. *CI* = [−.52, −.02] for combined samples of mentally healthy control participants and individuals with clinical BDD, *z* = −.23, *CI* = [−.30, −.15] for student and community samples, *z* = −.21, CI = [−.37, −.05] for the cosmetic surgery samples) compared to the uncorrected weighted partial correlations (*z* = −.16, *CI* = [−.36, .03] for clinical BDD samples, *z* = −.32, *CI* = [−.54, −.11] for combined samples of mentally healthy control participants and individuals with clinical BDD, *z* = −.20, *CI* = [−.27, −.14] for student and community samples, *z* = −.18, *CI* = [−.32, −.03] for the cosmetic surgery samples). BDD diagnosis emerged as a significant moderator in the meta-analysis of uncorrected zero-order correlations (cf., Table 2). More precisely, studies in which BDD was diagnosed by a clinician prior to or during study participation appeared to have higher negative correlations between BDD symptom severity and self-esteem compared to studies without clinician-rated BDD diagnoses. However, this was no longer significant in all other analyses (*b* = −.118, *CI* = [−.278, .043], *p* = .144 for corrected zero-order correlations). In an attempt to explore other factors which could explain the heterogeneity of effect sizes, we additionally conducted moderator analysis with the year of publication and examined differences between different measures of BDD symptom severity. None of these analyses had significant explanatory value.

**Table 2:** Moderator analyses of uncorrected zero-order correlations

Moderator	Level	Estimate	95%-CI	***p***-value
Mean age		−.006	[−.017, .006]	.316
Percentage of females		.003	[−.002, .008]	.208
Sample type^a^	BDD / HC	−.433	[−.729, −.138]	.006**
	Community / student	.008	[−.188, .205]	.931
	Cosmetic surgery	.003	[−.230, .235]	.980
Diagnostic method^b^	Self-report	.048	[−.120, .216]	.561
BDD diagnosis^c^	Yes	−.196	[−.357, −.035]	.019*

*Note:* Moderator analyses were conducted separately for each moderator. Intercepts were omitted in this table.

^a^ Dummy-coded with clinical BDD samples as the reference category

^b^ Dummy-coded with clinician-administered as the reference category

^c^ Dummy-coded with no as the reference category

* *p* < .05 **p < .01

### Publication bias

The funnel plots were rather symmetrical and did not point to any publication bias. Single effect sizes were positioned outside of the funnel which was in line with the heterogeneity of effect sizes, in particular with regard to the effect of the sample type. The funnel plots are attached as supplementary information (Additional files 3, 4, 5, 6).

## Discussion

We examined the relationship between BDD symptom severity and global self-esteem, while also investigating the role of depressive symptoms and other moderating factors. Regarding our three research questions, the following results were obtained: First, a moderate negative relationship between BDD symptom severity and self-esteem was revealed in meta-analyses of uncorrected and corrected zero-order correlations. Thus, the current state of research suggests that with increasing BDD symptoms self-esteem appears to be lowered. This is in line with previous findings from individual studies suggesting that BDD is often accompanied by low self-esteem (e.g., [3]). Thus, it appears negative evaluation in BDD is not limited to appearance but also extends to other domains of the self. Our results corroborate the role of appearance as an idealized value and dominating aspect in defining the self. Our results also provide an empirical basis for negative core beliefs (e.g., “I am worthless.”, “If my appearance is defective then I am worthless.”) that are often described as part of cognitive-behavioral models of BDD [62–64]. Furthermore, our findings are consistent with studies on other disorders that have also found a relationship between self-esteem and psychopathology [65].

Second, the negative relationship between BDD symptom severity and global self-esteem was only partly explained by depressive symptom severity. The meta-analyses of uncorrected and corrected partial correlations demonstrated that there was still a negative, though smaller, relationship beyond the influence of depressive symptoms. Thus, higher levels of BDD symptoms appear to be associated to lower levels of self-esteem even after controlling for depressive symptoms. This might be interpreted as a connection between appearance concerns and global self-esteem which is maintained after partialling out the distress and impairment due to depressive symptoms. It corresponds to findings on the association between body image or body dissatisfaction and self-esteem (e.g., [66, 67]). Moreover, the results could imply that individuals suffering from BDD symptoms and comorbid depressive symptoms might have particularly low self-esteem.

Third, the relationship between BDD symptom severity and self-esteem turned out to be stable across samples with varying mean age of participants and percentage of females. However, it should be noted that the mean age was rather young in most of the samples and the majority of samples consisted of more female than male participants. Consequently, there might have been too less variation to examine potential effects of these two moderators. Further, the overall effect size was robust regardless of the diagnostic method for the assessment of BDD symptom severity. This suggests that self-report and clinician-administered instruments for the assessment of BDD symptoms were equally capable of capturing the effect. With regard to the sample type, the combined samples of individuals with clinical BDD and mentally healthy control participants showed high negative uncorrected correlations compared to moderate negative uncorrected correlations for the other sample types. However, estimates of corrected correlations were more similar across samples types. Particularly for combined samples of individuals with BDD and mentally healthy controls the correlation was reduced after artifact correction, whereas it was noticeably raised in clinical BDD samples. This suggests that the effect of the sample type was caused by variance restriction and enhancement and not by actual differences between the sample types. Regarding the mean weighted partial correlations, effect sizes for the different sample types were very much aligned after artifact correction. The significant effect of the moderator BDD diagnosis on the uncorrected zero-order correlations might suggest that samples which included participants with diagnosed BDD tended to demonstrate higher negative correlations than student or community samples without clinical diagnostics. However, as this effect was much smaller and not significant for the corrected correlations, it is likely that range restriction/enhancement artifacts also contributed to this finding.

We observed substantial variations in effect sizes with regard to the meta-analyses of zero-order correlations. One explanation for this heterogeneity may be the influence of depressive symptom severity on the relationship between BDD symptom severity and self-esteem. The mean weighted partial correlations which were smaller than the mean weighted zero-order correlations and the substantially reduced amount of heterogeneity in the meta-analyses of partial correlations support this explanation. Other moderators that we considered to possibly have an impact on the systematic variation of effect sizes seemed to be not relevant or only in the context of a statistical artifact caused by relative range restrictions/enhancements. Since the included studies did not provide sufficient information on comorbidities, personality disorders, or medication, these variables could not be investigated. Also, we were not able to examine associated factors such as insight. Furthermore, cultural aspects might play a role and could not be controlled for in the analyses. For instance, the study by Ahmadpanah et al. [13] stands out with a correlation between BDD symptom severity and global self-esteem of only *r* = −.04. This study was conducted in an Iranian sample in which according to the authors the face, hair, and body shape are often covered and not visible for others [13]. Thus, cultural effects need to be considered when trying to understand the relationship between BDD symptoms and self-esteem. Further, the use of social media or bullying experiences might also act as moderators and their impact should be clarified in future studies.

### Limitations

The present meta-analysis has several limitations. First, we included studies using detailed clinician-administered measures of BDD symptom severity as well as shorter self-report screening instruments. These are of course not equally valid in assessing BDD symptom severity. For example, self-report measures might fail to differentiate BDD symptoms from preoccupation about actual defects (e.g., acne, scars) or weight-based concerns in the context of an eating disorder. Four of the 14 studies which applied self-report BDD measures tried to control for eating disorders. One of these studies excluded participants with elevated symptoms of an eating disorder [56], one study assessed comorbidities and reported that none of the participants were suffering from a comorbid eating disorder [6], one study excluded participants with a past or present eating disorder according to self-report [22], and one study ruled out the presence of any mental disorder according to self-report [11]. In order to address this limitation, we investigated the influence of the diagnostic method in moderator analysis. The diagnostic method appeared to have no systematic influence on the magnitude of effect sizes. On the one hand, this could imply that self-report measures were equally capable of capturing the relationship between BDD symptoms and self-esteem. On the other hand, this could signify that a preoccupation with actual appearance defects or weight-based concerns show a similar association with global self-esteem. Since this is the first meta-analysis on BDD and self-esteem we preferred to include all studies assessing BDD symptoms and self-esteem and controlled for the diagnostic method in moderator analysis.

Second, concerning the assessment of global self-esteem, this meta-analysis relied on the Rosenberg Self-esteem Scale [4] and considered the level of self-esteem only. Thus, we cannot determine whether other definitions and operationalizations of self-esteem demonstrate the same pattern of results. We were not able to examine contingencies and instability of self-esteem and their associations with BDD symptoms, since most of the primary studies did not assess these aspects of self-esteem.

Third, no causal inference can be drawn from our correlational findings. It remains unclear whether low self-esteem represents a vulnerability for BDD or develops as a consequence of the disorder (cf., [3]). Orth and Robins described different models for linking low self-esteem to depression [20] and these models might also apply to the relationship between BDD and self-esteem. Apart from unidirectional pathways, reciprocal relations or a common cause (e.g., bullying experiences) of both variables are possible. Moreover, a diathesis-stress model might be appropriate in which only under certain conditions low self-esteem leads to elevated BDD symptoms. Also, if low self-esteem predisposed BDD symptoms, mediating (e.g., social avoidance) and moderating variables (e.g., instability of self-esteem) might have an effect. Schulte et al. investigated the temporal dynamics of insight, affect, and self-esteem in BDD over six consecutive days and found that the cross-lagged effect of state self-esteem on insight was stronger than the effect of insight on state self-esteem [68]. Altogether, more studies are required to investigate causal directions.

Fourth, we included studies with varying ranges of BDD symptom severity. This may have led to overestimation of effect sizes for extreme group comparisons and underestimation of the effect in clinical samples. We tried to adjust effect sizes using variance corrections. However, in the absence of standard deviation norms for the individual BDD measures in the general population, we used standard deviation estimates from community samples if these were available or had to rely on student samples. Therefore, the results of the meta-analysis of corrected correlations have to be interpreted with caution, and the corrections need to be regarded as an imperfect attempt to deal with the heterogeneous samples.

Fifth, we were only able to exploratively investigate moderators for which sufficient information was provided in the studies. For instance, we could not control for effects of medication, comorbidities, or personality disorders. Hence, moderator analysis should be replicated in the future with a larger number of studies and variability of moderators.

### Future directions

Future studies may examine causal directions concerning the relationship between BDD symptom severity and self-esteem. Furthermore, future research may seek to identify subgroups in which BDD symptoms are associated with particularly low self-esteem, as these groups might benefit from self-esteem interventions. In this regard, it could be important to consider different developmental phases and the impact of depressive symptoms. It might also be helpful to examine whether low self-esteem can help to distinguish individuals with BDD from individuals without BDD among cosmetic surgery patients. Moreover, future studies should focus on different aspects of self-esteem. For instance, Buhlmann et al. investigated implicit self-esteem [5, 6], whereas B. Phillips et al. examined contingent self-esteem in BDD [10]. More research on contingencies and stability of self-esteem in BDD is required. With regard to prevention and therapy of BDD, an important step will be to evaluate the specific effects of interventions targeting self-esteem. In their network analysis of BDD and major depressive disorder Summers et al. revealed a high centrality of feelings of worthlessness and discussed implications for treatment such as addressing maladaptive core beliefs about self-worth [18]. Hence, future work may further try to determine the role of feelings of worthlessness in etiology, maintenance, and treatment of BDD. Furthermore, future trials may compare the effects of interventions intended to boost self-esteem and enhance self-compassion. In particular, focusing on self-compassion may entail certain benefits because it appears to be independent of personal achievements and success and thereby may result in more stable self-evaluations and reduced processes of comparing oneself to others (e.g., in the domain of appearance) [69]. Higher levels of self-compassion were associated with fewer BDD symptoms in a sample of adolescents [70]. Veale and Gilbert proposed to improve current treatments for BDD by developing a functional and evolutionary understanding of the BDD symptoms and by learning to relate to oneself and others with compassion and kindness [71]. These strategies from compassion-focused therapy [72, 73] might complement or enhance cognitive approaches.

## Conclusions

Altogether, our findings demonstrate that low self-esteem appears to be an important feature in BDD, particularly when not controlling for depressive symptoms. Consequently, addressing self-esteem and corresponding core beliefs is of high importance in the treatment of BDD. This emphasizes the value of cognitive restructuring and interventions such as the self-esteem pie by which one tries to reduce the overidentification with appearance and develop a more balanced basis of one’s self-esteem [63]. In this regard, a study by Rosen and Reiter found that decreases in BDD symptoms (as measured by the BDDE) after cognitive-behavioral therapy were associated with improvements in self-esteem [34]. Furthermore, depending on whether low self-esteem acts as a risk factor or as a consequence of BDD, self-esteem interventions might play a crucial role in the prevention of BDD. Low self-esteem during adolescence predicted adult psychopathology in a longitudinal birth cohort development study [74]. Consequently, BDD prevention programs might benefit from interventions targeted at cognitive and social determinants of low self-esteem (cf., [75]). This might buffer against the development of a negative bias in evaluating oneself which appears to be present in adolescents with high appearance anxiety [19]. Taken together, our results show that BDD is characterized by low self-esteem and highlight the importance of interventions targeting low self-esteem.

## Supplementary Information

**Additional file 1.** PRISMA Checklist.

**Additional file 2.** Standard deviation estimates for the BDD symptom severity measures used for artifact correction.

**Additional file 3.** Funnel plot for the meta-analysis of uncorrected zero-order correlations.

**Additional file 4.** Funnel plot for the meta-analysis of artifact-corrected zero-order correlations.

**Additional file 5.** Funnel plot for the meta-analysis of uncorrected partial correlations.

**Additional file 6.** Funnel plot for the meta-analysis of artifact-corrected partial correlations.

## Data Availability

The extracted data used for the meta-analysis are available at our Open Science Framework (OSF) data repository (https://osf.io/z52fc/).

## Abbreviations

BDD: Body dysmorphic disorder
DSM-IV: 4th edition of the Diagnostic and Statistical Manual of Mental Disorders
DSM-5: 5th edition of the Diagnostic and Statistical Manual of Mental Disorders
BDD-YBOCS: Yale-Brown Obsessive Compulsive Scale for Body Dysmorphic Disorder
BDDE: Body Dysmorphic Disorder Examination
FKS: Body Dysmorphic Symptoms Inventory (Fragebogen körperdysmorpher Symptome)
QDC: Questionario sul Dismorfismo Corporeo
DCQ: Dysmorphic Concern Questionnaire
BDDQ: Body Dysmorphic Disorder Questionnaire
BDD-DM: Body Dysmorphic Disorder Diagnostic Module
RSES: Rosenberg Self-Esteem Scale
BDI: Beck Depression Inventory
HAMD: Hamilton Depression Rating Scale
DASS: Depression subscale of the Depression Anxiety Stress Scales
HADS: Depression subscale of the Hospital Anxiety and Depression Scale
SCL-90: Depression subscale of the Symptom Checklist-90
PHQ-9: Patient Health Questionnaire-9 Depression module
CENTRAL: Cochrane Central Register of Controlled Trials
ICTRP: WHO International Clinical Trials Registry Platform
BDDE-SR: Body Dysmorphic Disorder Examination - Self Report
