# Supplementary material for: Body dysmorphic disorder and self-esteem: a meta-analysis
Source: BMC Psychiatry. 2021 Jun 15;21:310. doi: 10.1186/s12888-021-03185-3 (PMC8207567; doi:10.1186/s12888-021-03185-3)
Supplement: Supplementary file 2 — Additional file 2. Standard deviation estimates for the BDD symptom severity measures used for artifact correction. [file 12888_2021_3185_MOESM2_ESM.pdf]

Additional File 2. Standard deviation estimates for the BDD symptom severity measures used for artifact correction.

BDD measure	Standard deviation used for artifact correction	Source	Sample description
BDD-YBOCS, clinician-administered and self-report	8.86[a]	Phillips B, Moulding R, Kyrios M, Nedeljkovic M, Mancuso S. The relationship between body dysmorphic disorder symptoms and self-construals. Clin Psychol. 2011;15(1):10–6.	Community and student sample (n=194)
BDDE / BDDE-SR	28.26	Boroughs MS, Krawczyk R, Thompson JK. Body dysmorphic disorder among diverse racial/ethnic and sexual orientation groups: prevalence estimates and associated factors. Sex Roles. 2010;63(9):725–37.	Undergraduate students (n=1041)
FKS	9.85	Möllmann A, Dietel FA, Hunger A, Buhlmann U. Prevalence of body dysmorphic disorder and associated features in German adolescents: a self-report survey. Psychiatry Res. 2017;254:263–7.	Adolescents and young adults, 96.1% high school students (n=308)
QDC	38.46	Cerea S, Bottesi G, Grisham JR, Ghisi M. Body dysmorphic disorder and its associated psychological and psychopathological features in an Italian community sample. Int J Psychiatry Clin Pract. 2018;22(3):206–14.	Community sample with 59.35% students (n=615)

DCQ	4.70	Schmidt J, Martin A. Appearance teasing and mental health: gender differences and mediation effects of appearance-based rejection sensitivity and dysmorphic concerns. Front Psychol. 2019;10:579.	Community sample with 42.7% students (n=501)

Note: We tried to use standard deviation estimates from large community samples. If these were not available, we used estimates from student samples or from other (shorter/self-report) versions of the BDD measures. BDD-YBOCS, Yale-Brown Obsessive-Compulsive Scale Modified for Body Dysmorphic Disorder; BDDE, Body Dysmorphic Disorder Examination; BDDE-SR, Body Dysmorphic Disorder Examination-Self Report; FKS, Fragebogen körperdysmorpher Symptome; QDC, Questionario sul Dismorfismo Corporeo; DCQ, Dysmorphic Concern Questionnaire.

[a] A standard deviation of 7.38 was observed in this study. As this study applied only 10 out of 12 items from the BDD-YBOCS, the standard deviation was multiplied by 1.2 for artifact correction.
