# Supplementary figures and images for: Body dysmorphic disorder and self-esteem: a meta-analysis
Source: BMC Psychiatry. 2021 Jun 15;21:310. doi: 10.1186/s12888-021-03185-3 (PMC8207567; doi:10.1186/s12888-021-03185-3)

**Additional File 3. Funnel plot for the meta-analysis of uncorrected zero-order correlations.**

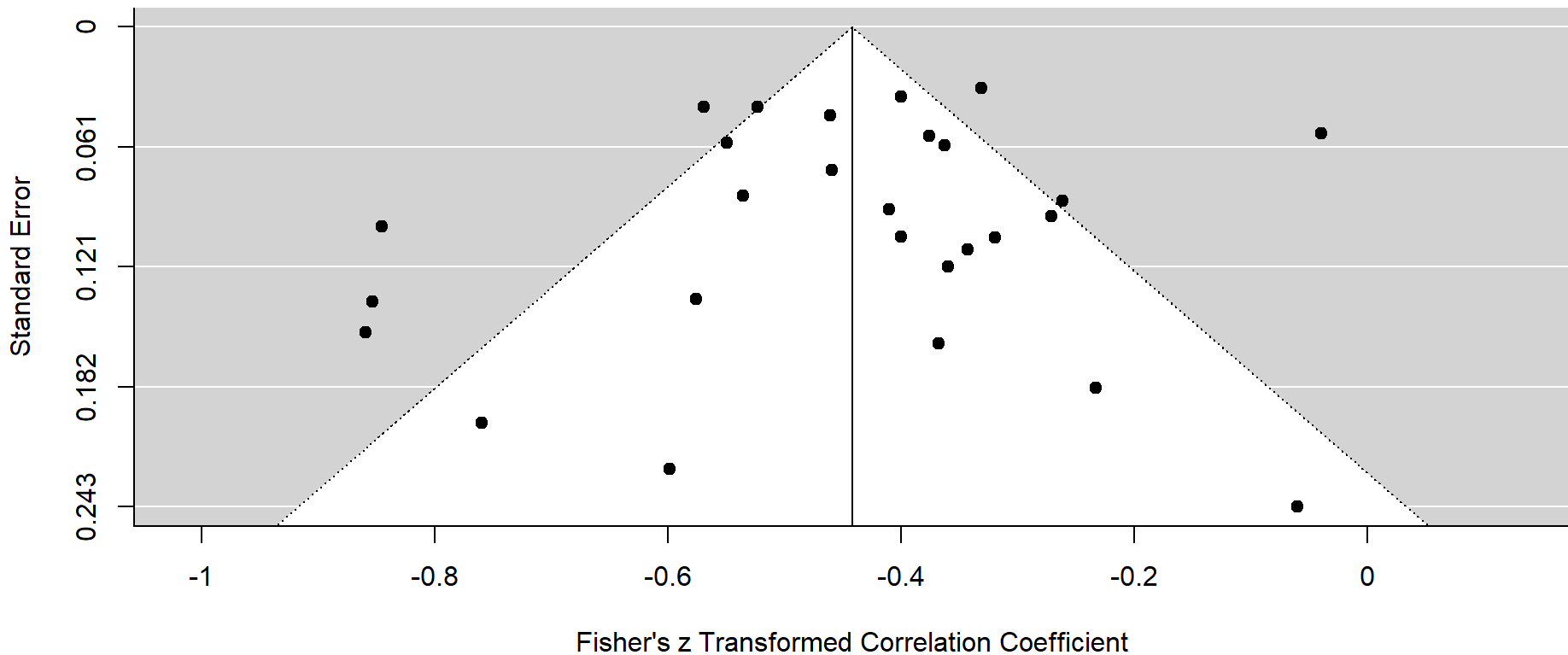

Supplement: Supplementary file 3 — Additional file 3. Funnel plot for the meta-analysis of uncorrected zero-order correlations. [file 12888_2021_3185_MOESM3_ESM.pdf]

**Additional File 5. Funnel plot for the meta-analysis of uncorrected partial correlations.**

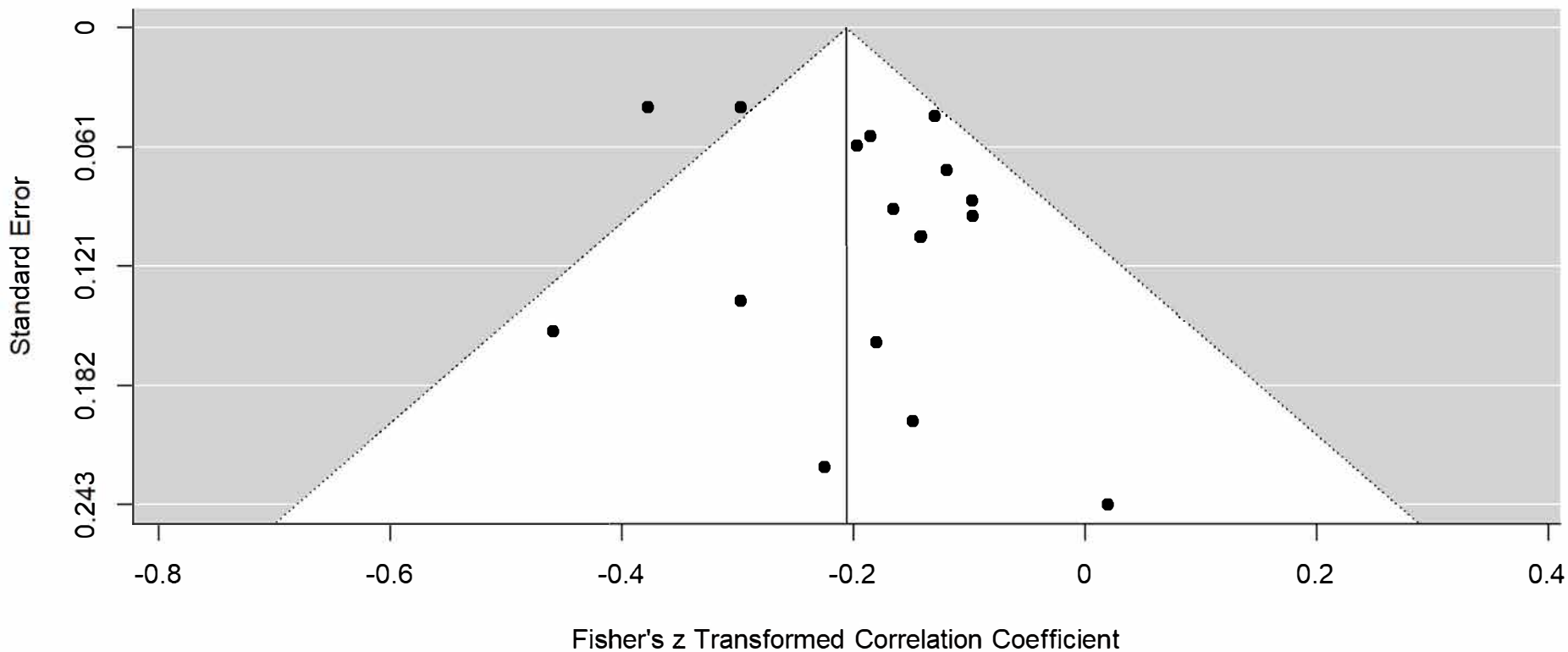

Supplement: Supplementary file 5 — Additional file 5. Funnel plot for the meta-analysis of uncorrected partial correlations. [file 12888_2021_3185_MOESM5_ESM.pdf]
