# Supplementary material for: Body dysmorphic disorder and self-esteem: a meta-analysis
Source: BMC Psychiatry. 2021 Jun 15;21:310. doi: 10.1186/s12888-021-03185-3 (PMC8207567; doi:10.1186/s12888-021-03185-3)

Additional File 4. Funnel plot for the meta-analysis of artifact-corrected zero-order correlations.

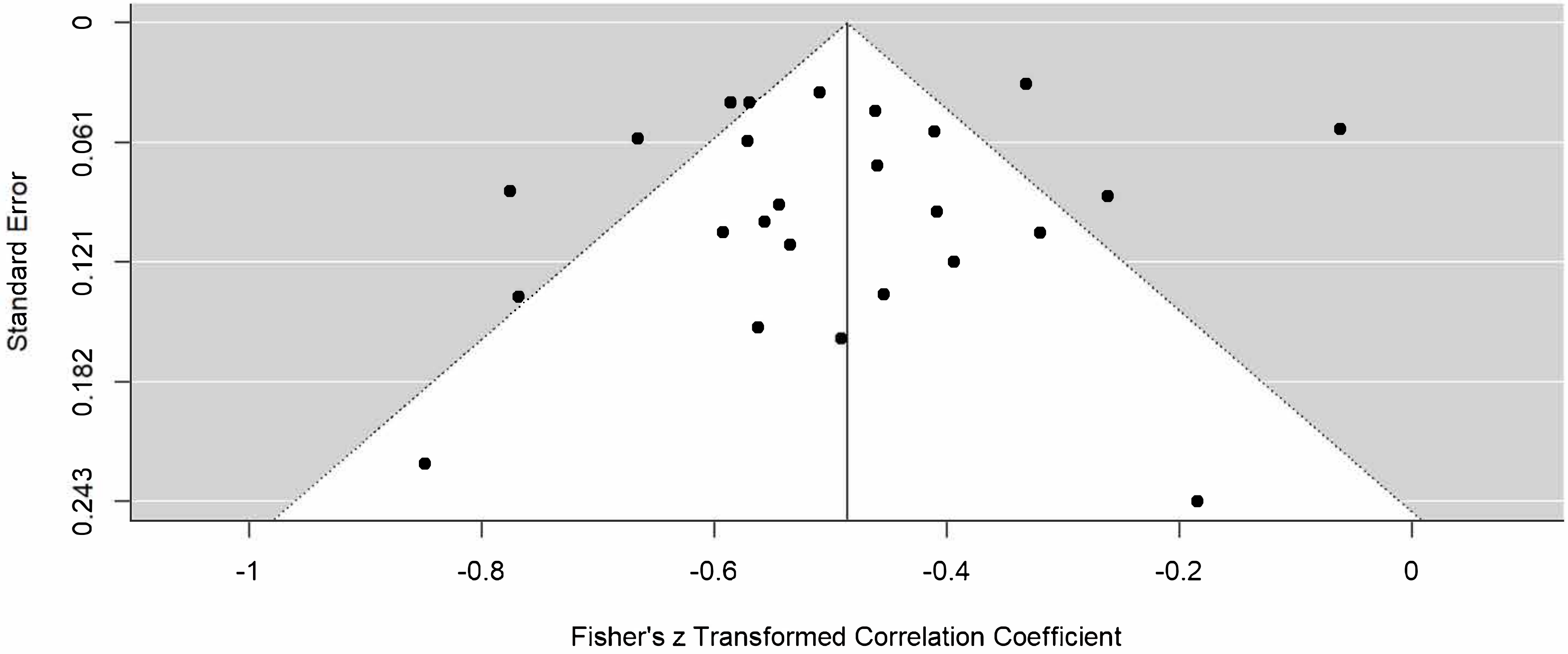

Supplement: Supplementary file 4 — Additional file 4. Funnel plot for the meta-analysis of artifact-corrected zero-order correlations. [file 12888_2021_3185_MOESM4_ESM.pdf]
